# New guidelines for systematic reviews and farewell to interviews

**DOI:** 10.1590/2177-6709.24.4.007-008.edt

**Published:** 2019

**Authors:** Flavia Artese, Carlos Flores-Mir

**Affiliations:** 1 Universidade do Estado do Rio de Janeiro, Departamento de Odontologia Preventiva e Comunitária (Rio de Janeiro/RJ, Brazil).; 2 University of Alberta, School of Dentistry, Division of Orthodontics (Edmonton, Canada).

The job of an Editor-in-Chief of a scientific journal is not restricted to facilitate the workflow of manuscript submission, assess reviewer’s suggestions and make final approval/rejection decisions. In addition to this, there is also a large amount of managing tasks, some of them dedicated to improve the positioning of the journal in the rank of publications of its specialty which involves bibliometric indexes. For the regular reader, this does not seem to make much difference, being nothing more than a series of bibliometric jargon. On the other hand, for the academic community, it is extremely important, since publishing in a higher impact journal is associated to a greater chance of visibility and prestige for a paper. In addition, some academic institutions use this ranking to link them to performance bonus and also with a greater chance of obtaining research-related funds.

In 2017, the *Dental Press Journal of Orthodontics* reached its peak in citation growth and this can be due to three reasons: (1) there was an adjustment in publication flow, reducing the publication time, and the number of articles in each volume was decreased from 137 (in 2013) to 78 (in 2016); (2) the DPJO was indexed in PubMed, which automatically made it visible to researchers all over the world; and (3) because of this, there was naturally an increase in the number of citations^1^. 

In 2018, we maintained the number of published papers per issue and, since the DPJO has fixed sections (Orthodontic Insight, Interview, Special Topic, Case Report by the Brazilian Board of Orthodontics and seven Original Articles), we analyzed each of them with an eye to citation potential. During the last 24 years, the DPJO interviewed 135 professionals who have contributed significantly to orthodontics. Although it is a well-read section and offers a historical contribution, it has not attracted adequate citations during its existence. With an eye in improving the DPJO bibliometric indexes, the Dental Press publishers have transferred this section to the *Revista Clínica de Ortodontia Dental Press* (a clinical orthodontic journal also indexed in Scopus).

In addition, we believe that acceptance of high-quality systematic reviews (SR) with or without meta-analysis would positively impact knowledge translation among readership. With this in mind, we have invited Dr. Carlos Flores-Mir to be the Associate Editor of a section on systematic reviews and meta-analyses. With the exponential amount of new research material published every year and the limited amount of time that clinicians and academicians have to read, SRs provide a nice synthesis of what is known for specific topics. Nevertheless, publications of SRs would not have the same frequency as the number of issues of this journal, therefore this section will be published whenever an adequate SR is accepted. Changes have been made in the Instructions for Authors, to reflect the high quality expected for SR submissions. There are dozens of SRs already published in orthodontics and repetition of topics or answering questions with low clinical importance will not be given any priority.

SRs are defined as a review that has been prepared using a predefined clear criterion for selecting articles, thereby minimizing biases^2^. In addition, these selected articles are assessed for risk of bias and consideration is given, at the end, to the certainty level of the stated conclusion. SRs have become the cornerstone of evidence-based health care. Publications of SRs in orthodontics have increased significantly in recent years, providing the orthodontic community up-to-date evidence regarding a particular question^2^ ^,^ ^3^. Up to 2000, there were no SR published in the orthodontic literature^2^, whereas in the period from 2000 to 2014, 157 SRs were identified in the leading orthodontic journals (American Journal of Orthodontics and Dentofacial Orthopedics, Angle Orthodontist, European Journal of Orthodontics, Journal of Orthodontics, and Orthodontics and Craniofacial Research)^3^. The preferred topics were: Class II treatment, followed by treatment mechanics and by oral hygiene and fluoride supplementation in orthodontics^3^.

Despite the increasing number of SRs being published in the orthodontic literature nowadays, both the quality of reporting^4^, as well as the quality of evidence used is still low to very low^3^. With the purpose of improving the quality of published SR, most orthodontic journals have implemented reporting guidelines, notably the PRISMA guidelines, which will also be implemented in the DPJO.

PRISMA stands for “Preferred Reporting Items for Systematic Reviews and Metanalyses” and comprises 27 items. Briefly, these items include: description of a protocol for the review, reporting at least one complete electronic search, assessment of risk of bias in and across included studies, description of selective outcome reporting, reporting of limitations of the review and of future research implications, and comment on sources of funding^4^. In addition, nowadays there is a clear expectation that the certainty level supporting the SR conclusions is adequately framed with the use of tools, such as the GRADE assessment tool.

Improving the quality of original research, and especially those of SR, that have a direct impact on clinical decision making, is a commitment of any scientific journal with society. There is even some argument suggesting that we are publishing more SRs than actual clinically impactful primary studies. So, DPJO is still committed and will give preference to primary clinical research, since they are the pillars for stronger SRs.

Good reading!
